# What is the association between the presence of comorbidities and the appropriateness of care for low back pain? A population-based medical record review study

**DOI:** 10.1186/s12891-018-2316-z

**Published:** 2018-11-06

**Authors:** Shanthi Ramanathan, Peter Hibbert, Louise Wiles, Christopher G. Maher, William Runciman

**Affiliations:** 1 Hunter Medical Research Institute, Locked Bag 100, New Lambton Heights, Newcastle, 2305 Australia; 2 Faculty of Health and Medicine, University of Newcastle, Newcastle, Australia; 3 Centre for Healthcare Resilience and Implementation Science, Australian Institute of Health Innovation, Macquarie University, Sydney, Australia; 4 Centre for Population Health Research, Sansom Institute for Health Research, The University of South Australia, Adelaide, Australia; 5 Institute for Musculoskeletal Health, Sydney, Australia; 6 Sydney School of Public Health, Sydney Medical School, University of Sydney, Sydney, Australia; 7 Australian Patient Safety Foundation, Adelaide, Australia

**Keywords:** Low back pain, Comorbidity, Appropriate care

## Abstract

**Background:**

Although “non-specific” in 90% of cases, low back pain (LBP) is often treated as an independent entity, even though comorbidities are commonly associated with it. There is evidence that some LBP may be related to chronic conditions or be a symptom of poor health. The purpose of this study was to clarify the extent of comorbidities amongst a cohort of Australian adults with LBP and examine if having concurrent conditions has any association with appropriateness of care for LBP.

**Methods:**

A population-based sample of patients with one or more of 22 common conditions was recruited by telephone; consents were obtained to review their medical records. Trained surveyors extracted information from their medical records to examine the care patients received for their LBP with respect to ten indicators of appropriate care, ratified by LBP experts. Using LBP as the index condition, lists of self-reported comorbidities and those that were documented in medical records were compared. Medical records were reviewed and analysed with respect to appropriateness of care to identify any significant differences in care received between patients with *LBP only* and those with LBP plus comorbidities.

**Results:**

One hundred and sixty four LBP patients were included in the analysis. Over 60% of adults with LBP in Australia had one of 17 comorbidities documented, with females being more likely than males to have comorbid conditions (63% vs 37%, *p* = 0.012). The more comorbidities, the poorer their reported health status (63% vs 30%, *p* = 0.006). Patients with comorbidities were significantly less likely to receive appropriate LBP care on nine of the ten LBP indicators (*p* < 0.05).

**Conclusions:**

This study established that the presence of comorbidities is associated with poorer care for LBP. Understanding why this is so is an important direction for future research. Further studies using a larger cohort are needed to explore the association between comorbidities and appropriateness of care for LBP, to better inform guidelines and practice in this area.

**Electronic supplementary material:**

The online version of this article (10.1186/s12891-018-2316-z) contains supplementary material, which is available to authorized users.

## Background

Low back pain (LBP) is the leading cause of activity limitation and absence from work, with over 70% of adults experiencing it at some stage in their lives [1, 2]. LBP is the health condition that has imposed the greatest disease burden globally since 1990 [3]. The direct costs of LBP have been estimated at AUD $4.8 billion per year in Australia [4]. However, these are minor compared with the indirect costs, which have been estimated at over AUD $8 billion per year in Australia [5].

Low back pain is often treated as an independent disorder with respect to the search for causes and cures. While the approach to LBP has, in recent years, moved from treating it as a purely anatomic-physiologic condition to a more complex multifaceted physical, neurochemical, biomechanical and psychosocial condition; the focus often continues to be on LBP as an isolated disorder [6]. However, comorbidities seem to be common with LBP [7–10], indicating that at least some back problems may not be distinct entities, but one of a number of symptoms of poor health in general.

In medicine, comorbidity is the presence of one or more additional diseases or disorders co-occurring with (that is, concurrent with) a primary disease or disorder and the rate of comorbidity and the number of chronic diseases experienced increases with age [11]. In Australia, almost 1 in 3 (29%) people aged 65 and over reported having three or more chronic diseases, compared with just 2.4% of those under 45 [10]. For a patient, comorbidities may have profound implications as the degree of physical and social disability rise with the number of co-existing conditions, which present several challenges in care [11–13]. Comorbidities are known to be associated with higher mortality and reduced quality of life and health providers need to take comorbid diseases into account when treating patients [6]. It is also suggested that future studies on consequences of comorbidity should investigate specific disease combinations [14].

As early as 1974, Gyntelberg showed significant relationships between LBP, angina pectoris, and various other diseases [15]; Biering-Sorensen (1984) found several physical conditions to be important indicators for future LBP [16]. Seferlis (2000) found a 4-fold increase in sick leave for other disorders in LBP patients [17] and Cote et al. (2007) noted that individuals seeking care for neck or back pain have worse health status than those who do not seek care [18]. A critical review of the LBP literature from the inception of Medline until the year 2000 found 23 separate studies that showed positive associations between LBP and the following disorders: headache/migraine, cardiovascular disease, respiratory disorders, neck pain, gynaecological disease, asthma, hay fever and other allergies, as well as general poor health [6]. According to the authors “*the literature leaves no doubt that diseases cluster in some individuals and that low back pain is part of this pattern*”. [6]

The National German Health Survey (*n* = 7124) found that all 31 physical diseases investigated were more common in subjects with LBP than those without LBP and that the most common were musculoskeletal disorders like rheumatoid arthritis, osteoarthritis and osteoporosis, followed by cardiovascular and cerebrovascular disease [8]. A Norwegian study found that LBP patients were significantly more likely to suffer from neck pain, upper back pain, pain in feet during exercise, headache, migraine, sleep problems, heat sensations, anxiety, and depression than patients without LBP [9]. In addition to physical disorders, both episodic and chronic LBP have also been shown to be significantly associated with mental illnesses such as depression, GI disease and anxiety [6, 19, 20] and increased healthcare utilization and costs [21]. Based on findings from the Australian Bureau of Statistic’s 2014–2015 National Health Survey (ANHS), LBP is featured in the second and third most common comorbidities in Australian adults, based on eight selected chronic diseases (i.e. arthritis, asthma, LBP, cancer, cardiovascular disease, chronic obstructive pulmonary disease, diabetes, and mental health conditions) [10]. However, little is known about the consequences of such comorbidities with respect to appropriateness of care for LBP.

The CareTrack Australia study was designed to establish baseline estimates of the appropriateness of care delivered, at a population level, for 22 common conditions [22]. The study used retrospective medical record review to assess compliance against a set of ratified indicators of appropriate care for each condition of which LBP was one. The compliance results for LBP have been published at indicator level and show an overall compliance of 72%. [23]. The objective of this paper is to use the larger CareTrack dataset with contains information on concurrent conditions for each patient to identify:

1. comorbidities amongst patients with LBP in the CareTrack sample
2. the conditions most likely to be associated with LBP
3. any associations between comorbidities and other socio-demographic variables included in the study
4. any associations between comorbidities and the appropriateness of LBP care for the associated conditions in Australian adults.

## Methods

**Fig. 1:**
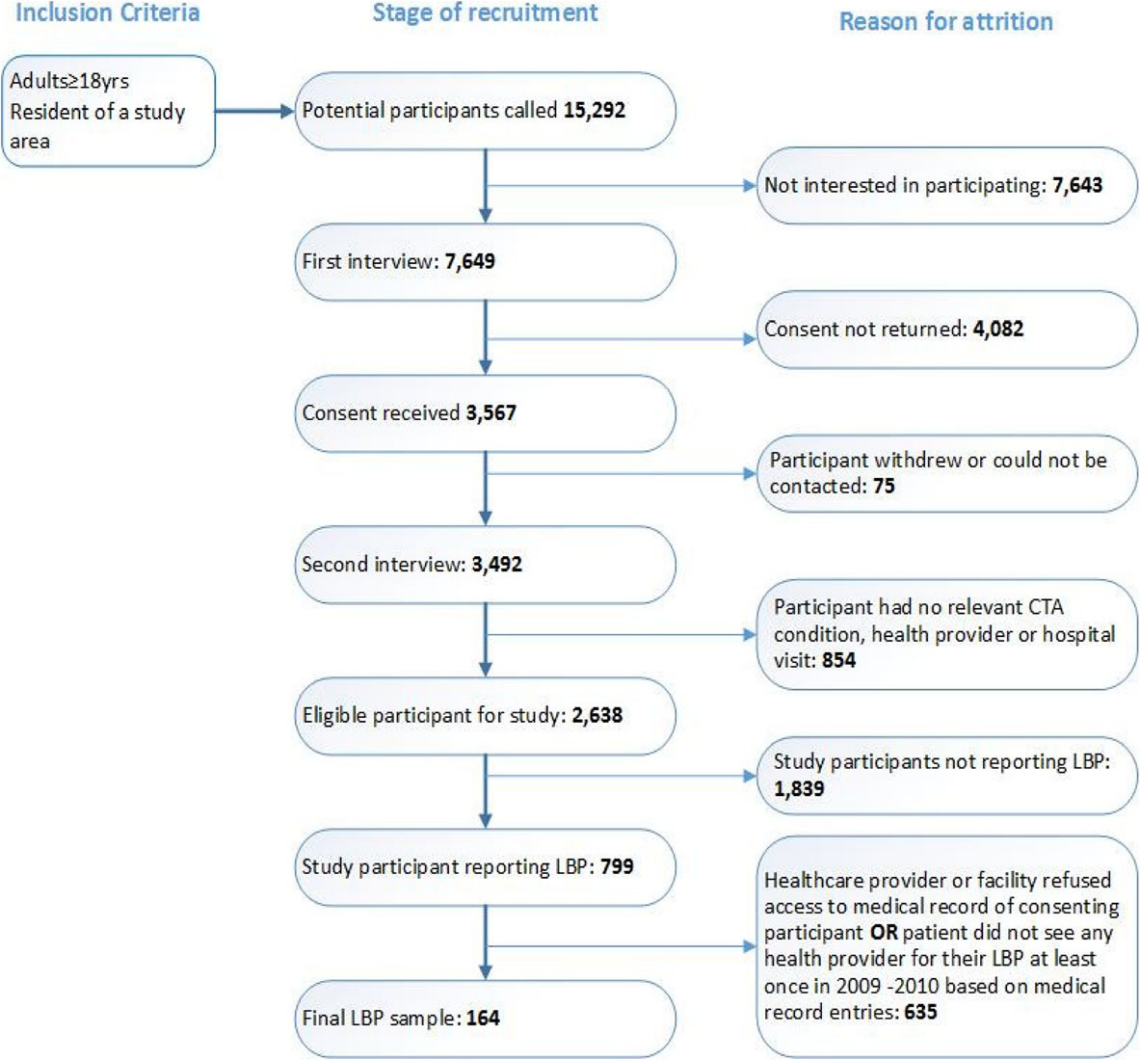
Inclusion criteria, stages of recruitment, and reasons for attrition from the CareTrack study

The CareTrack methods have been described in detail elsewhere. [22, 24] Here, we describe some aspects of particular relevance to an examination of comorbidities in the context of LBP. A sample of adults designed to be representative of the Australian population was randomly selected from a telephone directory (the Australian Telstra White Pages) from defined regions within two states New South Wales and South Australia. [25] One adult was randomly selected from each household and recruited over the phone. Those who agreed were subsequently sent a mail package containing information about the study and a consent form to allow access to their medical records. Participants who provided consent were contacted by phone and asked if they had one or more of the 22 CareTrack conditions, and which healthcare providers they had seen for these in the years 2009 and 2010. Healthcare providers including GPs, chiropractors, physiotherapists, and specialist physicians were contacted and asked for consent for trained CareTrack surveyors to access the medical records of consenting participants. Only participants who had documented care for LBP during 2009 and 2010 were included in this analysis. Human Research Ethics Committee approval was obtained to undertake the data collection from the University of South Australia and all relevant bodies and sites. Figure 1 provides a summary of the various stages of the recruitment protocol, the inclusion criteria, and attrition at each stage to obtain the final LBP sample.

Details of comorbidities in the CareTrack sample were captured in two ways. First, participants who consented to having their medical records accessed as part of CareTrack were asked to identify the conditions that they were treated for in 2009/2010 during the second phone call (see Additional file 1 for details of the question). Second, consenting participants for whom access to medical records was granted also had any relevant conditions noted (where present) from their medical records. The data obtained from both these methods are presented in this paper to identify comorbidities in the CareTrack LBP sample, however, as there were differences in the patterns found using the two methods only data from the patient records were used in the analysis for associations with socio-demographic variables and indicators of appropriateness of LBP care. The levels of comorbidity associated with LBP were collapsed to three groups: those without any comorbidities, those with one or two other conditions on the CareTrack list, and those with three or more of these.

Chi-square tests were run to determine if there were any significant differences in the socio-demographic characteristics and the appropriateness of care received by these three groups of LBP patients. Significant differences were deemed to be those at the 95% level of confidence (*p* > 0.05).

## Results

### Level of comorbidity amongst LBP patients

In response to the question *In 2009 and 2010 have you been treated for [CareTrack condition]?* over 80% of LBP patients in the CareTrack sample reported receiving treatment in 2009–2010 for at least one of the other 17 CareTrack conditions^1^ during their second phone interview. Table 1 shows that the number of patient-reported conditions in the LBP CareTrack sample ranged between 1 and 10. The mean number of patient-reported conditions was 3.27 (including LBP), and the mode and median were both 3. However, in contrast, only 60% of LBP patients had been treated for at least one other CareTrack condition in the years 2009 and 2010 according to the evidence in their medical record. Thus, the level of comorbidity amongst LBP patients based on evidence in their medical record was lower than the level of comorbidity self-reported by patients. Whereas only 19% of LBP patients reported not having been treated for any of the other conditions covered by the CareTrack study, 40% were treated for only LBP in 2009–2010, based on the information in their medical records. The mean number of conditions based on medical record data was 2.46, the mode was 1 and the median was 2.

**Table 1:** Patients reporting other conditions in addition to their LBP, self-report vs medical record

Number of patient-reported conditions including LBP	Self-reported conditions		Conditions in medical record	
	Number n	Percentage %	Number n	Percentage %
LBP only	31	18.9	63	38.4
2	28	17.1	33	20.1
3	38	23.2	28	17.1
4	34	20.7	18	11.0
5	13	7.9	11	6.7
6	14	8.5	8	4.9
7	2	1.2	3	1.8
8	1	0.6		
9	1	0.6		
10	2	1.2		
Total	164	100	164	100

*LBP* low back pain

### Conditions associated with LBP

The top ten specific comorbid conditions reported by LBP patients are presented in Table 2 with hypertension and hyperlipidaemia being the two most frequently reported (approximately two out of every five LBP patients reported having at least one of these conditions). Osteoarthritis and dyspepsia were the next most frequently reported conditions, with at least one in four LBP patients reporting that they had been treated for these conditions. The two most common comorbidities found in the medical records were hypertension (a condition for which approximately 29% of LBP patients had received treatment) and osteoarthritis (one in four LBP patients). For all conditions, except for obesity which was not asked of patients during their phone interview, there were far more patients who reported being treated for those conditions than there was evidence for in the medical record.

**Table 2:** Top ten comorbid conditions associated with LBP, based upon self-report and medical records

Top ten	Self-report		Medical record	
Conditions on the CareTrack list	Number of LBP patients with condition	Percentage of LBP patients with condition	Number of LBP patients with condition	Percentage of LBP patients with condition
Hypertension	67	40.9	47	28.7
Osteoarthritis	50	30.5	42	25.6
Hyperlipidaemia	66	40.2	26	15.9
Dyspepsia	42	25.6	22	13.4
Depression	36	22.0	16	9.8
Osteoporosis	25	15.2	13	7.9
Coronary Artery Disease	16	9.8	12	7.3
Diabetes	20	12.2	9	5.5
Asthma	23	14	8	4.9
Obesity (^a^)			15	9.1
Atrial Fibrillation	13	7.9		

Legend: (^a^) Not asked for during phone interviews, *LBP* low back pain

Data from the medical records were used to undertake the analysis on the association between the levels of comorbidity and patient socio-demographic factors and appropriateness of LBP care. Table 3 shows the number of patients by comorbidity level and the percentage in each group based on evidence found in their medical records.

**Table 3:** Recoding of LBP patients into three groups by number of comorbid conditions

Number of comorbidities	Found in medical records for 2009/2010	
	Number	Percentage(%)
LBP only	63	38.4
1–2 additional conditions	61	37.2
3 or more other conditions	40	24.4
Total	164	100.0

Legend: *LBP* low back pain

### Comorbidity and patient socio-demographic variables

Table 4 presents the data for associations between the seven socio-demographic variables included in CareTrack and the level of comorbidity of LBP patients. Asterisks indicate statistically significant differences in sociodemographic variables by level of comorbidity. Patients with no comorbidities were significantly more likely to be *male* than *female* (60% versus 40%) and those with one or more comorbidities were more likely to be *female* (63% versus 37%). This result indicates that in the CareTrack sample, females with LBP were more likely to have other chronic conditions than males*.*

**Table 4:** Patient comorbidities associated with other LBP patient variables

Demographic variable	Categories	LBP only (*n* = 63)	1–2 comorbidities (*n* = 61)	3 or more comorbidities (*n* = 40)	*p* value
Gender*	Male	60%	37%	37%	0.012*
	Female	40%	63%	63%	
Age	18–39	10%	8%	5%	0.574
	40–54	30%	29%	18%	
	55–74	52%	48%	58%	
	75+	8%	16%	18%	
Educational attainment	Year 10 & under	25%	25%	45%	0.421
	HSC	16%	14%	16%	
	Trade Cert/Diploma	40%	43%	26%	
	University	19%	17%	13%	
Work status	Employed	44%	31%	34%	0.186
	Unemployed	2%	0%	3%	
	Retired	48%	52%	58%	
	Student/other	6%	18%	5%	
Health status*	Poor to fair	30%	35%	63%	0.006*
	Average	33%	40%	26%	
	Good to excellent	37%	25%	11%	
Accessibility of healthcare	Difficult to very difficult	14%	14%	21%	0.575
	Neither	8%	11%	16%	
	Easy to very easy	78%	75%	63%	
Medical literacy	Low	3%	3%	8%	0.669
	Moderate	16%	13%	18%	
	High	81%	84%	74%	

Legend: * = statistically significant difference, *p* < 0.05, *LBP* low back pain

The second significant difference was that LBP patients who had three or more comorbidities were significantly more likely to report *poor to fair* health (63%) compared with those who had 1–2 other conditions (35%) and those who had *LBP only* (30%). Similarly, those with *LBP only* were significantly more likely to report *very good to excellent* health (37%) compared with only 11% of those with three or more comorbidities. There were no significant associations between level of comorbidity and age, educational attainment, work status, accessibility of healthcare or medical literacy.

### Comorbidity and appropriate LBP care

Compliance with CareTrack indicators for appropriateness of care is shown in Table 5. Analyses revealed statistically significant differences in rates of compliance by number of comorbidities for nine of the ten LBP indicators for appropriateness of care assessed as part of the CareTrack study (see Table 5).

**Table 5:** Compliance by comorbidities, medical record data

Indicator	Overall Compliance % (95% CI)	LBP Only (%)	1–2 comor-bidities (%)	3 or more comor-bidities (%)	*p* value
Medical history documented at presentation	94 (92–96)	96.9	90.1	87.2	< 0.001*
Physical examination performed and documented at presentation	87% (84–90)	95.0	76.5	64.4	< 0.001*
Assessed for spine fractures (trauma, history of previous fracture, prolonged use of steroids)	81% (77–84)	86.1	71.2	75.0	< 0.001*
Assessed for cancer (history of cancer, unexplained weight loss, immunosuppression)	75% (71–79)	81.2	64.9	65.8	< 0.001*
Assessed for infection (fever, IV drug use)	41% (37–46)	48.8	26.5	34.9	< 0.001*
Neurological examination performed – (strength, sensation and reflexes in lower limbs)	63% (58–67)	66.9	55.9	43.9	< 0.001*
Assessed for cauda equina syndrome	50% (45–54)	53.2	49.8	31.8	< 0.001*
NOT prescribed any of the following medications: dexamethasone, other oral steroids, colchicine or antidepressants	83% (75–92)	97.1	83.9	67.6	0.005*
DID NOT receive any of the following treatments: transcutaneous electrical nerve stimulation (TENS), lumbar corsets and support belts, spinal traction	94% (89–99)	95.0	93.4	94.4	0.933
NOT advised to rest in bed.	98% (89–100)	100.0	100.0	91.4	0.003*

Legend: * = statistically significant difference, *p* < 0.05, *LBP* low back pain

For the first six indicators, a clear pattern is evident. Patients who had been treated for one or more “other” conditions besides LBP in 2009–10 were significantly less likely to have had (i) their medical history documented, (ii) a physical or neurological examination, or (iii) assessments for infection, (iv) assessment for cancer or (v) assessment for fractures than patients who had *LBP only*. In addition, compared with patients with *LBP only* and *those with fewer than three comorbidities* those patients with *three or more comorbid conditions* were also significantly less likely to be assessed for cauda equina syndrome, more likely to be prescribed dexamethasone, other oral steroids, colchicine or antidepressants and more likely to not be advised against resting in bed*.* The only indicator that was not associated with comorbidity was the one relating to NOT receiving transcutaneous electrical nerve stimulation (TENS), lumbar corsets and support belts or spinal traction (*p* = 0.933).

## Discussion

Understanding more about comorbidities can provide vital information for prevention, management and treatment of diseases including LBP. This study found that at least 62% of the 164 LBP patients in the CareTrack study had at least one other chronic condition. Comparisons with data from the 2014–15 ANHS [10] and the first German National Health Survey [9] are summarised in the bottom half of Table 6 (results). The CareTrack proportion of LBP sufferers with comorbidities obtained from their medical record was similar to the ANHS findings (62% versus 65%) but was significantly higher when using their survey data (81% vs 65%). In contrast, the German national study sample [8] had significantly higher rates of comorbidity compared with both the CareTrack medical record (62%) and survey (81%) proportions. Rather than reflect an inconsistency in the findings, these discrepancies are most likely a consequence of the different methods used in each study, as summarised in the top half of Table 6. The two most significant of these in terms of impact on comparability are: (1) the way LBP and other conditions were defined and (2) the number of conditions included in each study. Both the ANHS and the German study asked participants if they had “ever” had a range of conditions whereas the CareTrack study asked them if they had “*been treated for* [condition] *in the years 2009/2010*.” In addition, definitions of LBP ranged from having LBP within a 7-day period to having it for over 6 months. Second, the number of conditions covered by the three studies varied from eight to 31. These differences made comparability of prevalence of comorbidity problematic.

**Table 6:** Comparability of CareTrack findings and methodological features with the ANHS and the German National Survey

		CareTrack Medical Records	CareTrack Survey	National Health Survey [10]	German Health Survey [8]
Methods	Based on self-report	N	Y	Y	Y
	Country	Australia	Australia	Australia	Germany
	Year data collected	2011	2011	2016	1997–1999
	Inclusion criteria (LBP)	Treated for condition in 2009–10	Condition lasted less than 6 months (acute)	Lifetime prevalence and had condition for more than 6 months (chronic)	Suffered from LBP in past 7-days – 7 day prevalence
	Inclusion criteria (other)	Treated for condition in 2009–10	Treated for condition in 2009–10	Lifetime prevalence and had condition for more than 6 months (chronic)	Ever been diagnosed - lifetime prevalence
	No. of comorbid conditions included	17	17	8	31
Results	LBP only	38%	19%	35%	8%
	LBP plus 1 condition	20%	17%	30%	13%
	LBP plus 2 or more conditions	42%	64%	35%	77%
	Proportion with comorbidities	62%	81%	65%	92%
	Highest comorbid condition	Hypertension (28.7%)	Hypertension (40.9%)	Osteoarthritis (31.4%)	Osteoarthritis (50%)
	2nd highest	Osteoarthritis (25.6%)	Hyperlipidaemia (40.2%)	Circulatory system disease (30.9%)	Gastritis (30%)
	3rd highest	Hyperlipidaemia (15.9%)	Osteoarthritis (30.5%)	Mental and Behavioural disorder (29.8%)	Hypertension (26%)

Legend: 10 = Australian Institute of Health and Welfare. Australia’s health 2016. Australia’s Health Series No 15. Catalogue No AUS 199. AIHW. Canberra; 2016. 8 = Schneider S, Mohnen SM, Schiltenwolf M, Rau C. Comorbidity of low back pain: Representative outcomes of a national health study in the Federal Republic of Germany. European Journal of Pain. 2007;11(4):387–97

In contrast, the most prevalent conditions to be associated with LBP showed similarity across all three studies, despite some definitional issues. Hypertension, osteoarthiritis and hyperlipidaemia were the three most prevalent conditions for LBP patients in the CareTrack study and the first two were also ranked top three in the other studies, confirming that LBP is most closely associated with cardiovascular and musculoskeletal conditions.

Hypertension and hyperlipidaemia were also consistent with more than 23 other studies that clearly illustrate that LBP is associated with cardiovascular disease and poor health overall [8]. A potential reason for this association may be that these diseases share some common risk factors such as physical inactivity and overweight/obesity. Together these findings support the theory by Hestbaek and colleagues [6] that LBP is often clustered with other conditions and poorer overall health. This clustering of conditions (with LBP as the index condition) is more prevalent amongst females, a finding consistent with the ANHS data and the German study – both found that females (with or without LBP) had significantly more health problems than their male counterparts [8, 10]. Patients with three or more comorbidities were also more likely to report *poor to fair* health compared with those who had 1–2 other conditions or those without comorbidities, confirming previous findings that LBP is associated with a person’s health status which would be expected to deteriorate with each additional chronic condition.

A significant finding from this CareTrack study sub-analysis was that LBP patients who had comorbid conditions were at greater risk of receiving care of a lower quality than those who only had LBP. This is the first time that this type of analysis has ever been undertaken for LBP patients. Even having one additional condition meant that LBP patients were significantly less likely to be adequately examined and assessed for a range of conditions such as fractures, cancer and infection and those with three or more comorbid conditions were at further risk of being prescribed unnecessary medication and not being given appropriate advice for managing their LBP. This finding has important implications for managing patients with LBP. Healthcare providers treating LBP need to ensure that patients with comorbidities are adequately examined, assessed and managed. This issue of appropriateness of care also needs to be examined in detail using a larger cohort to explore why patients with more co-morbidities are less likely to receive appropriate LBP care and to determine if particular disease combinations are at greater risk of sub-standard care. While current LBP guidelines appear generic enough to apply to all patients they should be tested on a range of patients with various comorbidities. [26]

Further studies should also examine continuity of care and patient satisfaction, important areas for patients with more than one disease who are likely to be treated by several healthcare providers simultaneously. Such studies can also focus on determining the nature of the relationship between LBP and other comorbidities – does LBP cause other diseases or vice versa; do these conditions simply co-exist or do they have a common cause or risk factors?

### Strengths and limitations

The key strength of the CareTrack methods is in the random selection of patients (a population-based rather than a convenience-based sample). However, an unavoidable consequence of this strategy, compounded by limited research funds, was that the number of participants with LBP was low (*n* = 164), especially when compared with similar studies. The approach used was also associated with a high rate of attrition of potential participants (see Fig. 1) and several sources of potential bias, particularly in favour of recruiting older Australians. Another potential limitation is the possibility that care had been provided but was not recorded, an issue estimated to affect about 5% but no more than 10% of instances. [27]. A final limitation is the number of conditions included in the study and confinement to conditions that were treated in 2009–10, both of which were likely to have reduced the overall level of comorbidity amongst the CareTrack LBP cohort. Within the scope of this study, it was not possible to definitively assess the accuracy of both patient self-reports and medical records. It is possible that patients may have had some of these other conditions they identified at some point in their lifetime (particularly in the period between 2010 and when they were interviewed) but not have received any treatment for them in 2009–2010, hence their absence from parts of the medical records reviewed. Equally plausible explanations for the discrepancy could be incomplete medical records and/or inaccurate patient recall.

## Conclusion

Findings from this sub-study of CareTrack data indicate that there is a moderate to high level of comorbidity amongst LBP patients in Australia and that comorbidity is more prevalent in females, consistent with previous studies. The findings also confirm prior evidence that LBP is associated with cardiovascular illness, other musculoskeletal conditions and poorer general health. The most significant finding from this study was that LBP patients with comorbidities were significantly less likely to receive appropriate care for LBP. Future studies using a larger cohort are needed to explore this association between comorbidity and appropriate care for LBP to better inform clinical guidelines and practice in this area.

## Additional file

Additional file 1: Question regarding comorbidities asked of CareTrack participants. (DOCX 12 kb)

## Abbreviations

ANHS: Australian National Health Survey
AUD: Australian Dollar
CareTrack: Care Track Australia Study
GI: Glycaemic index
LBP: Low back pain

## Footnotes

1 Some other conditions such as cancer, venous thromboembolism and surgical site infections, whilst part of the CareTrack study, were not assessed as part of the self-reporting component of the research.

## References

[1] Oliveira CB, Maher CG, Pinto RZ, Traeger AC, Lin C-WC, Chenot J-F, et al. Clinical practice guidelines for the management of non-specific low back pain in primary care: an updated overview. Eur Spine J. 2018. 10.1007/s00586-018-5673-2. 10.1007/s00586-018-5673-2 29971708

[2] Walker BF, Muller R, Grant WD (2004). Low back pain in Australian adults. Prevalence and associated disability. J Manip Physiol Ther.

[3] Vos T, Abajobir AA, Abate KH, Abbafati C, Abbas KM, Abd-Allah F (2017). Global, regional, and national incidence, prevalence, and years lived with disability for 328 diseases and injuries for 195 countries, 1990-2016: a systematic analysis for the global burden of disease study 2016. Lancet.

[4] Arthritis and Osteoporosis Victoria and Deloittes Access Economics. A problem worth solving: The rising cost of musculoskeletal conditions in Australia. In*.* Elsternwick: Arthritis and Osteoporosis. Victoria; 2013.

[5] Crow WT, Willis DR (2009). Estimating cost of care for patients with acute low back pain: a retrospective review of patient records. J Am Osteopath Assoc.

[6] Hestbaek L, Leboeuf-Yde C, Manniche C (2003). Is low back pain part of a general health pattern or is it a separate and distinctive entity? A critical literature review of comorbidity with low back pain. J Manip Physiol Ther.

[7] Beales DJ, Smith AJ, O'Sullivan PB, Straker LM (2012). Low Back pain and comorbidity clusters at 17 years of age: a cross-sectional examination of health-related quality of life and specific low Back pain impacts. J Adolesc Health.

[8] Schneider S, Mohnen SM, Schiltenwolf M, Rau C (2007). Comorbidity of low back pain: representative outcomes of a national health study in the Federal Republic of Germany. Eur J Pain.

[9] Hagen EM, Svensen E, Eriksen HR, Ihlebæk CM, Ursin H (2006). Comorbid subjective health complaints in low Back pain. Spine.

[10] AIHW, Australian Institute of Health and Welfare (2016). Australia’s. Health 2016. Australia's Health Series No 15. Catalogue No AUS 199.

[11] Newman AB (2012). Comorbidity and multimorbidity.

[12] Søndergaard E, Willadsen TG, Guassora AD, Vestergaard M, Tomasdottir MO, Borgquist L (2015). Problems and challenges in relation to the treatment of patients with multimorbidity: general practitioners’ views and attitudes. Scand J Prim Health Care.

[13] Gijsen R, Hoeymans N, Schellevis FG, Ruwaard D, Satariano WA, van den Bos GAM (2001). Causes and consequences of comorbidity: a review. J Clin Epidemiol.

[14] Gijsen R, Hoeymans N, Schellevis F, Ruwaard D, Satariano W, van den Bos G (2001). Causes and consequences of comorbidity. J Clin Epidemiol.

[15] Gyntelberg F (1974). One year incidence of low back pain among male residents of Copenhagen aged 40-59. Dan Med Bull.

[16] Biering-Sorensen F (1984). Physical measurements as risk indicators for low-Back trouble over a one-year period. Spine.

[17] Seferlis T, Németh G, Carlsson A-M (2000). Prediction of functional disability, recurrences, and chronicity after 1 year in 180 patients who required sick leave for acute low-Back pain. J Spinal Disord Tech.

[18] Coté M, Rossignol M, Dionne C, Truchon M, Arsenault B, Poitras S (2007). An interdisciplinary guideline development process: the clinic on low-back pain in interdisciplinary practice (CLIP) low-back pain guidelines.

[19] Nordin M, Hiebert R, Pietrek M, Alexander M, Crane M, Lewis S (2002). Association of comorbidity and outcome in episodes of nonspecific low back pain in occupational populations. J Occup Environ Med.

[20] Gore M, Sadosky A, Stacey BR, Tai K-S, Leslie D (2012). The burden of chronic low Back pain: clinical comorbidities, treatment patterns, and health care costs in usual care settings. Spine.

[21] Ritzwoller D, Crounse L, Shetterly S, Rublee D (2006). The association of comorbidities, utilization and costs for patients identified with low back pain. BMC Musculoskelet Disord.

[22] Runciman W, Hunt T, Hannaford N, Hibbert P, Westbrook J, Coiera E (2012). CareTrack: assessing the appropriateness of health care delivery in Australia. Med J Aust.

[23] Ramanathan SA, Hibbert PD, Maher CG, Day RO, Hindmarsh DM, Hooper TD (2017). CareTrack: toward appropriate Care for low Back Pain. Spine.

[24] Hunt Tamara D, Ramanathan Shanthi A, Hannaford Natalie A, Hibbert Peter D, Braithwaite Jeffrey, Coiera Enrico, Day Richard O, Westbrook Johanna I, Runciman William B (2012). CareTrack Australia: assessing the appropriateness of adult healthcare: protocol for a retrospective medical record review. BMJ Open.

[25] Telstra Corporation Limited (2010). Telstra White Pages.

[26] Uhlig K, Leff B, Kent D, Dy S, Brunnhuber K, Burgers JS (2014). A framework for crafting clinical practice guidelines that are relevant to the care and management of people with multimorbidity. J Gen Intern Med.

[27] McGlynn EA, Asch SM, Adams J, Keesey J, Hicks J, DeCristofaro A (2003). The quality of health care delivered to adults in the United States. N Engl J Med.

