# Supplementary material for: What is the association between the presence of comorbidities and the appropriateness of care for low back pain? A population-based medical record review study
Source: BMC Musculoskelet Disord. 2018 Nov 6;19:391. doi: 10.1186/s12891-018-2316-z (PMC6220516; doi:10.1186/s12891-018-2316-z)
Supplement: Supplementary file 1 — Question regarding comorbidities asked of CareTrack participants. (DOCX 12 kb) [file 12891_2018_2316_MOESM1_ESM.docx]

**Appendix 1 : Question regarding comorbidities asked of CareTrack participants**

| Clinical conditions | **Question: In 2009 and 2010 have you been treated for …... ?**  # Alcohol Dependence (Alcoholism)  # Asthma (Shortness of breath)  # Atrial Fibrillation (Heart arrhythmia, abnormal heart rhythm)  # Stroke/TIA (CVA/Trans Ischemic Attack)  # Community Acquired Pneumonia (Lung disease)  # Chronic Heart Failure  # Chronic obstructive pulmonary disease (Emphysema/Chronic bronchitis)  # Coronary Artery Disease (Heart Attack/Angina)  # Depression  # Diabetes (high blood sugar)  # Dyspepsia (acid reflux/stomach ulcer)  # Hypertension (high blood pressure)  # Hyperlipidemia (high cholesterol)  # Low Back Pain  # Obesity (overweight)  # Osteoarthritis  # Osteoporosis (brittle bones)  # Panic Disorder  **Response options:**  1. Yes  2. No  8. Unsure/Refused (check and supplement with Medicare/ data extraction) |
| --- | --- |
